# Pflegeheim-sensitive Krankenhausfälle und Ansätze zur Verringerung der Hospitalisierung von Pflegeheimbewohnerinnen und -bewohnern

**DOI:** 10.1007/s00103-022-03654-4

**Published:** 2023-01-10

**Authors:** Maria Paula Valk-Draad, Sabine Bohnet-Joschko, PSK-Studiengruppe, Maria Paula Valk-Draad, Katja Stahl, Christel Bienstein, Hans-Jürgen Heppner, Andreas Sönnichsen, Hagen Sjard Bachmann, Petra Thuermann, Oliver Gröne, Paula Zietzsch, Helmut Hildebrandt, Thomas Klie, Sabine Bohnet-Joschko

**Affiliations:** 1 Lehrstuhl für Management und Innovation im Gesundheitswesen, Fakultät für Wirtschaft und Gesellschaft, Universität Witten/Herdecke, Alfred-Herrhausen-Str. 50, 58448 Witten, Deutschland; 2 Lehrstuhl für Community Health Nursing, Fakultät für Gesundheit, Universität Witten/Herdecke, Witten, Deutschland

**Keywords:** Potenziell vermeidbare Krankenhausfälle, Handlungsempfehlung, Pflegeheim, Gesundheitsversorgung, Geriatrie, Potentially avoidable hospitalizations, Recommendation, Nursing home, Healthcare, Geriatrics

## Abstract

**Hintergrund:**

Interventionen zur Vermeidung von potenziell risikobehafteten Krankenhauseinweisungen aus dem Pflegeheim sind von hoher Bedeutung für Patientensicherheit und Versorgungsqualität. Ein Katalog Pflegeheim-sensitiver Krankenhausfälle (PSK) bildet die Grundlage für die Entwicklung von Handlungsempfehlungen.

**Methoden:**

In zwei vorangegangenen Forschungsphasen entwickelte ein Expertenpanel einen Katalog mit 58 PSK mittels eines angepassten Delphi-Verfahrens (die RAND/UCLA Appropriateness Methode). Dieses Verfahren wurde von der Nord-Amerikanischen gemeinnützigen Research and Development Organisation (RAND) und Klinikern der Universität von Kalifornien in Los Angeles (UCLA) entwickelt. In der hier vorgestellten dritten Projektphase wurden zunächst in einem Expertenworkshop Interventionsansätze zu deren Reduktion entwickelt. Die Ergebnisse wurden anschließend durch sechs Gutachtende aus themenverwandten Sektoren bewertet, ergänzt und systematisch zur Erstellung von Handlungsempfehlungen verwendet. Mögliche Umsetzungshürden wurden berücksichtigt und der Zeithorizont der Wirksamkeit abgeschätzt.

**Ergebnis:**

Die Handlungsempfehlungen betreffen Bereiche der Kommunikation, Kooperation, Dokumentation und Versorgungskompetenz sowie einrichtungsbezogene, finanzielle und rechtliche Aspekte. Einzelne Indikationsbündel demonstrieren die Relevanz für das deutsche Gesundheitswesen. Zur Erhöhung der Wirksamkeit empfehlen die Sachverständigen eine sinnvolle Kombination von Handlungsempfehlungen.

**Diskussion:**

Durch eine Optimierung multidisziplinärer Kommunikation und Kooperation, kombiniert mit einem – auch digitalen – Ausbau der Infrastruktur bei Schaffung einrichtungsbezogener rechtlicher Voraussetzungen und Vergütungsstrukturen, könnten fast 35 % aller Hospitalisierungen aus dem Pflegeheim, rund 220.000 Krankenhausfälle in Deutschland, vermieden werden. Die für Maßnahmen erforderlichen Ausgaben könnten aus Einsparungen durch vermiedene Krankenhausaufenthalte in Höhe von 768 Mio. € refinanziert werden.

**Zusatzmaterial online:**

Zusätzliche Informationen sind in der Online-Version dieses Artikels (10.1007/s00103-022-03654-4) enthalten.

## Abstract (en)

**Background:**

Interventions to reduce potentially risky hospitalizations among nursing home residents are highly relevant for patient safety and quality improvement. A catalog of nursing home-sensitive conditions (NHSCs) grounds the policy recommendations and interventions.

**Methods:**

In two previous research phases, an expert panel developed a catalog of 58 NHSCs using an adapted Delphi-procedure (the RAND/UCLA Appropriateness Method). This procedure was developed by the North American non-profit Research and Development Organisation (RAND) and clinicians of the University of California in Los Angeles (UCLA). We present the third phase of the project focused on the development of interventions to reduce NHSCs starting with an expert workshop. The workshop results were then evaluated by six experts from related sectors, supplemented, and systematically used to produce recommendations for action. Possible implementation obstacles were considered and the time horizon of effectiveness was estimated.

**Results:**

The recommendations address communication, cooperation, documentation and care competence as well as facility-related, financial, and legal aspects. Indication bundles demonstrate the relevance for the German healthcare system. To increase effectiveness, the experts advise a meaningful combination of individual recommendations.

**Discussion:**

By optimizing multidisciplinary communication and cooperation, combined with an– also digital – expansion of the infrastructure and the creation of institution-specific and legal prerequisites as well as remuneration structures, an estimated 35% of all hospitalizations, approximately 220,000 hospitalizations for Germany, could be prevented. The implementation expenditure could be refinanced by avoided hospitalization savings amounting to 768 million euros.

## Hintergrund

Der Altersdurchschnitt der Bevölkerung in Deutschland steigt weiter an und damit auch die Zahl der Pflegebedürftigen: 2020 erhielten ca. 4,3 Mio. Menschen Leistungen aus der sozialen Pflegeversicherung, von denen ca. 820.000 in Pflegeheimen versorgt werden; bis 2030 werden es bereits 5,1 Mio. Menschen sein (+ 19 %), und bis 2040 werden 5,7 Mio. Pflegebedürftige (+ 12 %) erwartet [1, 2]. Da Pflegebedürftigkeit zu einem großen Teil altersbedingt ist, steigt bei zunehmender Zahl der hochaltrigen Pflegebedürftigen auch die Anzahl der in Heimen gepflegten Personen [2–4].

Bei einer Verschlechterung des Gesundheitszustands von Pflegeheimbewohnerinnen und -bewohnern (PHB) kommt es in Deutschland pro Quartal bei 21 % [5], bzw. pro Jahr bei 44 % [6] der PHB mindestens einmal zu einer Krankenhauseinweisung, sodass sich mit dem Anstieg der PHB auch die Zahl der Hospitalisierungen erhöhen wird [7]. Für die akut erkrankten PHB besteht die Gefahr, dass sich ihr Gesundheitszustand durch Hospitalisierung weiter verschlechtert [8, 9], beispielsweise durch „Transferstress“ [10], unerwünschte Arzneimittelwirkungen [11, 12], „Post-Hospital-Syndrom“ [13] sowie im Krankenhaus erworbene Erkrankungen („hospital-acquired conditions“) [14]. Krankenhauseinweisungen von PHB können zu einem gewissen Teil als unangemessen und vermeidbar betrachtet werden [15, 16].

Zur Beurteilung potenziell vermeidbarer und evtl. risikobehafteter Krankenhausfälle unter PHB werden häufig die sogenannten ambulant-sensitiven Krankenhausfälle (ASK) herangezogen, die durch präventive Maßnahmen oder verbesserte Versorgung gegebenenfalls im ambulanten Sektor versorgt werden könnten. Listen mit ASK wurden zuerst für die USA und anschließend auch für das deutsche Gesundheitssystem entwickelt [17–19]. Entsprechend kann auch von „Pflegeheim-sensitiven Krankenhausfällen“ (PSK) gesprochen werden. Es gibt jedoch wesentliche Unterschiede zwischen ASK und PSK. Die Altersstruktur der PHB und die damit einhergehende Zahl der Komorbiditäten, das geriatrische/gerontologische Erkrankungsspektrum, der Heilungsprozess der älteren Bevölkerung, die erforderlichen medizinischen Maßnahmen und die Intensität der Versorgung im Pflegeheim zeigen deutliche Unterschiede zur ambulanten Versorgung auf [20, 21]. Bestehende ASK-Listen können daher nur bedingt Anhaltspunkte für die Versorgung akut erkrankter PHB geben [22]. In diesem Kontext wurde das Projekt „Pflegeheim-sensitive Krankenhausfälle“ an der Universität Witten/Herdecke in Kooperation mit OptiMedis AG, Pflege e. V. und FIVE e. V. entwickelt und durchgeführt. Es ist Teil der umfangreichen Forschung zur Vermeidung von Krankenhauseinweisungen in Deutschland (vgl. weitere über den Innovationsfonds^1^ geförderte Projekte [23–38]).

Für die Analyse wie auch für die Entwicklung von Handlungsempfehlungen ist der Praxisbezug zu Versorgungssystem und -setting entscheidend [39]. Die Implementationsforschung hat in diesem Kontext den Begriff des „valley of death“ geprägt, weil Ergebnisse, die unter gut kontrollierten Studienbedingungen erzielt wurden, sich in der Praxis als wenig effektiv erweisen bzw. wenig praxistauglich sind (Lücke zwischen Theorie und Praxis). Es wird daher empfohlen, relevante Stakeholder in alle Phasen der Forschung einzubeziehen, Kontextanalysen durchzuführen und Mixed-Methods-Studiendesigns unter Einbezug von interdisziplinären Teams den Vorzug zu geben [40].

Das PSK-Projekt wurde dementsprechend in einem sequenziellen Mixed-Methods-Design angelegt [41]. Der vorliegende Artikel präsentiert Ergebnisse der dritten Phase, in der Handlungsempfehlungen zur Reduktion von Hospitalisierungen kontextspezifisch, d. h. für das deutsche Pflegeheimsetting und unter Einbindung von Expertinnen und Experten aus den beteiligten Sektoren, entwickelt wurden [40]. Zuvor waren in einer ersten Phase die häufigsten Krankenhauseinweisungen aus dem Pflegeheim ermittelt worden (Häufigkeitsanteil ≥ 0,1 %). In einer zweiten Phase wurden daraus die PSK identifiziert, hier definiert als Diagnosen, die unter bestmöglichen Versorgungsbedingungen zu zumindest 70 % im Pflegeheim behandelbar wären. Diese ersten beiden Phasen zur Entwicklung eines PSK-Katalogs wurden mitsamt einer kritischen Reflexion der angewandten Methoden bereits an anderer Stelle ausführlich dargestellt ([6]; vgl. Kurzfassung im Methodenabschnitt). Um Handlungsempfehlungen gezielt für das Pflegeheimsetting und speziell für die PSK in Deutschland zu entwickeln, waren die Fragestellungen für die dritte Forschungsphase:

1. Welche strukturellen Voraussetzungen oder praktischen Konzepte und Maßnahmen haben das Potenzial zur Reduktion der PSK?
2. Was ist der geschätzte Zeithorizont ihrer Wirksamkeit?
3. Wie hoch sind die Präventionspotenziale für das System der gesetzlichen Krankenversicherungen (GKV) durch die Reduktion von PSK? Präventionspotenziale beziffern Ausgaben, die durch die Umsetzung von Handlungsempfehlungen schätzungsweise vermieden werden können.

## Methode

In einem sequenziellen Mixed-Methods-Design [41] wurden vorab 1. Sekundärdaten zur Bestimmung der Häufigkeit von und Ausgaben für Krankenhauseinweisungen aus dem Pflegeheim analysiert (April–Sept. 2019) und 2. ein PSK-Katalog mithilfe eines angepassten Delphi-Verfahrens („RAND/UCLA Appropriateness Method“) konsentiert (Okt. 2019–Sept. 2020). Basierend auf diesen ersten zwei Studienphasen [6], die anschließend zum besseren Verständnis beschrieben werden, wurden 3. spezifische Handlungsempfehlungen zur Reduktion dieser PSK unter PHB für Deutschland entwickelt (Okt. 2020–Dez. 2021; dieser Artikel).

In der ersten Phase der Forschung wurden gemäß dem Standard „Gute Praxis Sekundärdaten-Analyse“ [42] aggregierte und anonymisierte Routinedaten von sechs GKVen analysiert, um die häufigsten Krankenhausfälle aus dem Pflegeheim mit einem minimalen Häufigkeitsanteil von 0,1 % zu identifizieren sowie damit verbundene Ausgaben zu beziffern.

**Figure 1:**
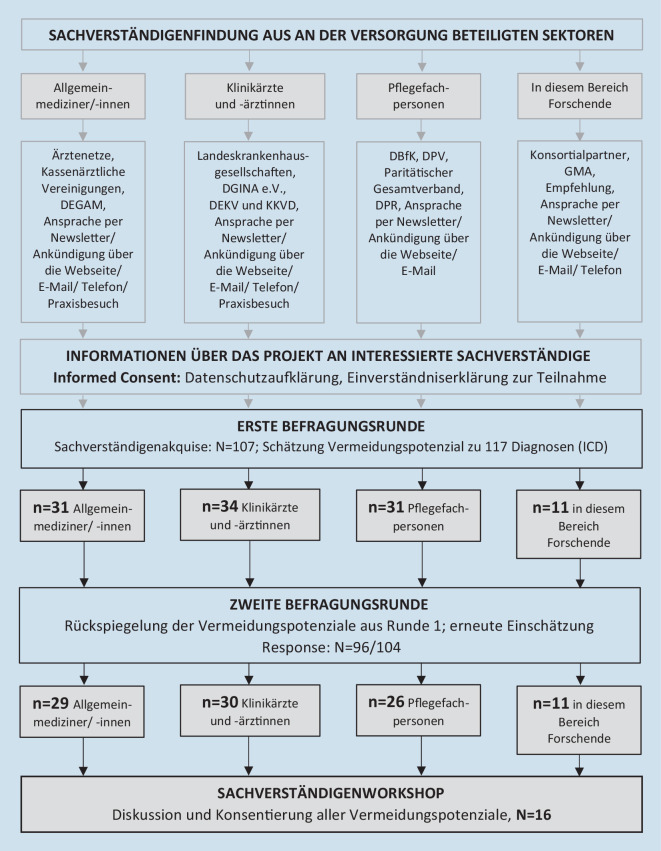

Die in Phase 1 identifizierten Diagnosen wurden dann in eine zweistufige Delphi-Befragung gegeben (Phase 2), mit 107 bzw. 96 Expertinnen und Experten in der ersten bzw. zweiten Befragungsrunde (31 bzw. 29 ambulant und 34 bzw. 30 klinisch tätige Ärztinnen und Ärzten, 31 bzw. 26 Pflegefachpersonen sowie 11 bzw. 11 Wissenschaftlerinnen und Wissenschaftlern), die durchschnittlich 16 Jahre Berufserfahrung hatten. Die Expertinnen und Experten wurden gebeten, erfahrungsbasiert einzuschätzen, welche tatsächliche Einweisungsdiagnose der angegebenen Entlassungsdiagnose in der Versorgungsrealität am ehesten zugrunde liegen könnte, um dann zu abstrahieren, welcher Anteil der Hospitalisierungen bei optimalen Versorgungsbedingungen sowie unter Berücksichtigung des Einflusses von Komorbidität und Schwere der Erkrankung ggf. vermeidbar wäre. Um zentrale Probleme bei der Abstrahierung der Vermeidungspotenziale vorab zu adressieren und vor allem das Vermeidungspotenzial der ungeplanten stationären Aufenthalte abzubilden, wurden dem Expertenpanel Hinweise und realitätsnahe Beispiele zur Verfügung gestellt, zu Themen wie Grundannahmen zu optimalen Versorgungsbedingungen, Umgang mit Behandlungspräferenzen und Patientenverfügungen, Krankenhaustermine für ambulante Initialversorgung, laufende (Chemo‑)Therapien und Diagnostik (vgl. Zusammenfassung der Hinweise im Onlinematerial auf S. 2–3; Volltexte in „Extended Data“ [43] zum PSK-Katalog [6]). Die Delphi-Befragung wurde dann gemäß der „RAND/UCLA Appropriateness Method“ [44] ergänzt um einen – wegen der SARS-CoV-2-Pandemie digital per ZOOM stattfindenden – Workshop mit einigen Mitgliedern des großen Expertenpanels sowie einigen mit dem Thema vertrauten weiteren Personen aus den Bereichen der GKV, Gesundheitsökonomie, Gesundheitswissenschaft sowie Gesundheitssystemforschung. Diese Vorgehensweise kombiniert die besten verfügbaren wissenschaftlichen Erkenntnisse mit dem kollektiven Urteil von Expertinnen und Experten, um zu einer Aussage über das Vermeidungspotenzial von PSK zu kommen, und wurde in ähnlicher Weise auch für die Entwicklung der ASK angewandt [19, 44]. Die Sachverständigenakquise sowie der RAND-UCLA-Prozess sind in Abb. 1 dargestellt. Das Sachverständigengremium konsentierte die PSK mit hohem (≥ 70 %) und konsistent eingeschätztem Vermeidungspotenzial als „Pflegeheim-sensitiv“.

Basierend auf der Einschätzung des rund 100-köpfigen Expertenpanels erarbeitete das gleiche Sachverständigengremium im zweiten Teil des Workshops Ansätze für die Versorgung akut erkrankter PHB (Phase 3). Die Workshop-Ergebnisse wurden während des per ZOOM digital stattfindenden Treffens mittels eines digitalen Whiteboards, in Freihandprotokollen sowie in vorbereiteten Protokollmatrizen dokumentiert. Sie fanden Eingang in sechs Gutachten (Allgemeinmedizin, Geriatrie, Pharmakologie, Gesundheitssystemforschung, Pflege, Rechtswissenschaft). In diesen wurden die Potenziale und Hürden der Vermeidung von PSK aufgezeigt, ggf. die Empfehlungen ergänzt bzw. Hinweise für die praktische Verwertbarkeit abgegeben, wobei sektorenübergreifende und systemverändernde Konzepte einbezogen wurden.

Abschließend wurden die Handlungsempfehlungen und die PSK strukturiert und systematisch in einer Matrix zusammengeführt. Unklarheiten wurden mithilfe der Sachverständigen geklärt und einzelne Empfehlungen sinnvoll zusammengelegt, um daraus Fallbeispiele abzuleiten. Gleichzeitig wurde auf der Grundlage von Krankenkassendaten das Präventionspotenzial für die Refinanzierung dieser Maßnahmen berechnet.

## Ergebnisse

### Identifizierung der Pflegeheim-sensitiven Krankenhausfälle und Präventionspotenzial

Zum besseren Verständnis der dritten Phase des PSK-Projektes werden zuerst kurz die wichtigsten Ergebnisse der ersten beiden Phasen geschildert. Darauf aufbauend wird das Präventionspotenzial dargestellt, d. h. die Schätzung der Ausgaben, die hochgerechnet für Deutschland vermieden werden könnten, wenn die Handlungsempfehlungen zur Optimierung der Versorgung der PHB umgesetzt wären.

Zur Analyse der Krankenhauseinweisungshäufigkeit und der damit assoziierten Kosten in Phase 1 des PSK-Projektes konnten die Angaben zu Krankenhauseinweisungen von 242.236 PHB herangezogen werden. Diese repräsentierten 30 % aller gesetzlich krankenversicherten PHB in Deutschland aus dem Jahr 2017. Wir identifizierten 117 Krankenhausentlassungsdiagnosen mit einem minimalen Häufigkeitsanteil von 0,1 %. Insgesamt 107 Expertinnen und Experten aus vier verschiedenen Fachbereichen schätzten in der ersten und 96 in der zweiten Delphi-Runde (effektive Responserate = 91 %) den Anteil der potenziell vermeidbaren Krankenhauseinweisungen für diese 117 Diagnosen ein (Phase 2 des PSK-Projektes). Die vier Fachbereiche des Expertenpanels der zweiten Befragungsrunde schätzten das Vermeidungspotenzial bei 83 von 117 Diagnosen ähnlich ein (vgl. Tab. 5 in [6]). Für die weiteren 34 Diagnosen lagen die Einschätzungen im Median 5 Prozentpunkte auseinander. Die Klinikärztinnen und -ärzte schätzten das Vermeidungspotenzial etwas niedriger und Wissenschaftlerinnen und Wissenschaftler etwas höher ein (Kruskall-Wallis-Test, *p* = 0,007). In einem anschließenden Sachverständigenworkshop (*n* = 16) wurden 25 Diagnosen mit hohem Vermeidungspotenzial diskutiert, die nicht konsistent eingeschätzt wurden (Streuung in den Einschätzungen von 75 % aller Expertinnen und Experten um den Median ≥ 15 %). 23 dieser Diagnosen wurden zusammen mit weiteren 35 Diagnosen mit hohem (≥ 70 %) und konsistent eingeschätztem Vermeidungspotenzial vom Sachverständigengremium als Pflegeheim-sensitiv konsentiert (Ergebnis Phase 2 des PSK-Projektes: 58 PSK).

In der online verfügbaren Supplementär-Tab. 1 sind die durch Präventionsmaßnahmen vermeidbaren Ausgaben für die einzelnen PSK – abgeleitet aus Ergebnissen der Projektphasen 1 und 2 – dargestellt: Das Vermeidungspotenzial beschreibt hier die Krankenhausfälle unter PHB, die laut Expertenpanel bei bestmöglicher Versorgung im Pflegeheim behandelt werden könnten. Dieser Anteil liegt z. B. für Herzinsuffizienz bei 75 %. Von den jährlich auftretenden Krankenhauseinweisungen für Herzinsuffizienz unter PHB, 33.524 aufgrund der für Deutschland hochgerechneten GKV-Daten, könnten somit 25.143 vermieden werden. Das Präventionspotenzial beziffert die durch Umsetzung von Handlungsempfehlungen vermeidbaren Ausgaben. Bei Krankenhausfallkosten für Herzinsuffizienz von ca. € 3683 errechnet sich ein Präventionspotenzial von ca. € 92,6 Mio.

Der Vergleich mit ambulant-sensitiven Krankenhausfällen [19] deckte Unterschiede auf: 53 % der 58 PSK waren nicht Teil der ASK und 69 % der ASK kamen nicht in den 117 unter PHB häufiger auftretenden Diagnosen (Häufigkeitsanteil ≥ 0,1 %) vor. Auch die Vermeidbarkeitspotenziale unterschieden sich: Für die Diagnosen, die dazu miteinander verglichen werden konnten, lagen Minimum und Maximum zwischen − 41 bis + 23 Prozentpunkte auseinander.

Die Ausgaben für einen Krankenhausaufenthalt eines PHB betrugen über alle PSK hinweg durchschnittlich ca. € 4030. Die Extrapolation der Häufigkeit und der durchschnittlichen Ausgaben auf den nationalen deutschen Kontext ergab, dass in Deutschland im Jahr 2017 etwa 2,6 Mrd. € für 646.000 Hospitalisierungen unter den knapp 820.000 PHB anfielen. Davon wären nach Einschätzung der PSK-Experten und -Expertinnen ca. 35 %, d. h. € 768 Mio., für ca. 220.000 Pflegeheim-sensitive Krankenhauseinweisungen potenziell vermeidbar [6].

In Tab. 1 sind die PSK nach anatomisch-therapeutischen Kategorien (Indikationsbündeln) zusammengefasst. Hinsichtlich PSK sind Harnwegserkrankungen zusammen mit Störungen des Volumenhaushalts unter PHB am häufigsten (8,4 % aller Krankenhausfälle, vgl. vorletzte Spalte). Herz-Kreislauf-Erkrankungen folgen mit 8,0 %, wobei allein die Herzinsuffizienz mit 5,2 % für zwei Drittel dieser PSK verantwortlich ist. Der Anteil der Atemwegserkrankungen (ohne Influenza-Grippe, ohne Pneumonien) ist nur halb so hoch. Unter Berücksichtigung von Pneumonien, die das Kriterium für PSK (Vermeidungspotenzial ≥ 70 %) verfehlten, rücken Atemwegserkrankungen mit einem Anteil von 12,2 % deutlich an die erste Stelle. Trotz unterdurchschnittlicher Fallkosten ist hier angesichts des hohen Fallaufkommens das zweithöchste Präventionspotenzial ausgewiesen (ca. € 167 Mio.). Neurologische Erkrankungen, inklusive demenzieller Erkrankungen, haben wegen der relativ hohen Fallkosten das höchste Präventionspotenzial (über € 192 Mio.), trotz eines eher moderaten Anteils von 6,8 %. An dritter und vierter Stelle folgen die Herz-Kreislauf-Erkrankungen mit knapp € 157 Mio. und – aufgrund der relativ niedrigen Fallkosten – die Harnwegserkrankungen/Volumenstörungen mit € 119 Mio.

**Table 1:** 

Indikationsbündel von PSK^b^ und ausgewählte weitere Indikationsbündel^c^ (betreffende ICD^d^)	Gesamtanzahl der Fälle für Deutschland im Jahr	Davon potenziell vermeidbare PSK-Fälle für Deutschland	Gesamtausgaben für Deutschland im Jahr	Präventionspotenzial für Deutschland im Jahr	Kumulativer Krankenhausfälle-Anteil in %	Durchschnittliche Fallkosten
Herz-Kreislauf-Erkrankungen (I10, I50, I70, I80, I95)	51.982	40.408 (78 %)	202.465.768 €	156.784.789 € (77 %)	8,0	3895 €
Neurologische Erkrankungen, exkl. demenzielle Erkrankungen (F05, F06, F07, F10, F20, F32, F33, G35, G40)	36.797	30.196 (82 %)	196.552.822 €	157.674.581 € (80 %)	5,7	5342 €
Demenzielle Erkrankungen (F01, G20, G30)	7324	6270 (86 %)	40.354.498 €	34.657.344 € (86 %)	1,1	5510 €
Harnwegserkrankungen und Störungen des Volumenhaushalts (N18, N30, N39) bzw. (E86, E87)	54.498	43.976 (81 %)	147.284.982 €	119.147.643 € (81 %)	8,4	2703 €
Magen-Darm-Erkrankungen (A04, A08, A09, K21, K25, K26, K29, K52, K57, K59, K62, R11)	36.595	29.839 (82 %)	100.806.716 €	81.029.398 € (80 %)	5,7	2755 €
Diabetes mellitus Typ 2 (E11)	10.719	9647 (90 %)	44.989.542 €	40.490.588 € (90 %)	1,7	4197 €
Respiratorische Erkrankungen, exkl. Pneumonien und Influenza (J20, J22, J40, J44)	28.555	23.136 (81 %)	100.763.967 €	80.221.777 € (80 %)	4,4	3529 €
*Pneumonie *(**NICHT PSK**^c^) *(J15, J18, J69)*	*50.674*	*22.306* *(44* *%)*	*198.257.299* *€*	*86.984.642* *€* *(44* *%)*	*7,8*	*3912* *€*
Dermatologische Erkrankungen (A46, C44, L02, L89)	11.779	9597 (81 %)	53.641.533 €	44.388.223 € (83 %)	1,8	4554 €
Muskuloskelettale Erkrankungen und Verletzungen (M54, R26, S00, S01, S20, S30, S70, S80)	18.042	15.611 (87 %)	31.189.786 €	26.888.419 € (86 %)	2,8	1729 €
*Frakturen *(**NICHT PSK**^c^) *(M80, S02, S22, S32, S42, S52, S72, S82)*	*50.806*	*4463* *(9* *%)*	*307.710.320* *€*	*22.980.990* *€* *(7* *%)*	*7,9*	*6057* *€*
*Sepsis *(**NICHT PSK**^c^) *(A40, A41)*	*15.944*	*0* *(0* *%)*	*79.219.677* *€*	*0* *€* *(0* *%)*	*2,5*	*4968* *€*
Sonstige PSK: Influenza-Grippe (J10); Hals- und Brustschmerzen (R07); Anämien (D50, D64); Kauapparat (K08); Dysphagie (R13); Cataracta senilis (H25)	13.117	10.765 (81 %)	29.916.435 €	24.418.546 € (82 %)	2,0	2281 €

Sämtliche Angaben zu den Hochrechnungen für Deutschland basieren auf den Daten der sechs gesetzlichen Krankenkassen bezogen auf alle Pflegeheimbewohnerinnen und -bewohner in Deutschland im selben Zeitraum (818.289 Pflegeheimbewohnerinnen und -bewohner zu den 242.236 unserer Stichprobe)

^a^Weitere Informationen zu allen PSK sind in der Supplementär-Tab. 1 aufgenommen

^b^PSK: Pflegeheim-sensitiver Krankenhausfall

^c^NICHT PSK: Einige ICD-10-GM-Dreisteller haben die Kriterien für PSK verfehlt (z. B. einige Pneumoniediagnosen (z. B. J15, J18) sowie Sepsis (z. B. A40, A41)). Aufgrund des relativ hohen Fallanteils sind sie für die Praxis von Bedeutung. Hier sind sie in kursiver Schrift dargestellt, um sie von den PSK abzuheben

^d^ICD: 10. Edition der International Classification of Diseases, deutsche Version, Dreisteller (ICD-10-GM-Dreisteller)

### Handlungsempfehlungen zur Reduktion Pflegeheim-sensitiver Krankenhausfälle

**Figure 2:**
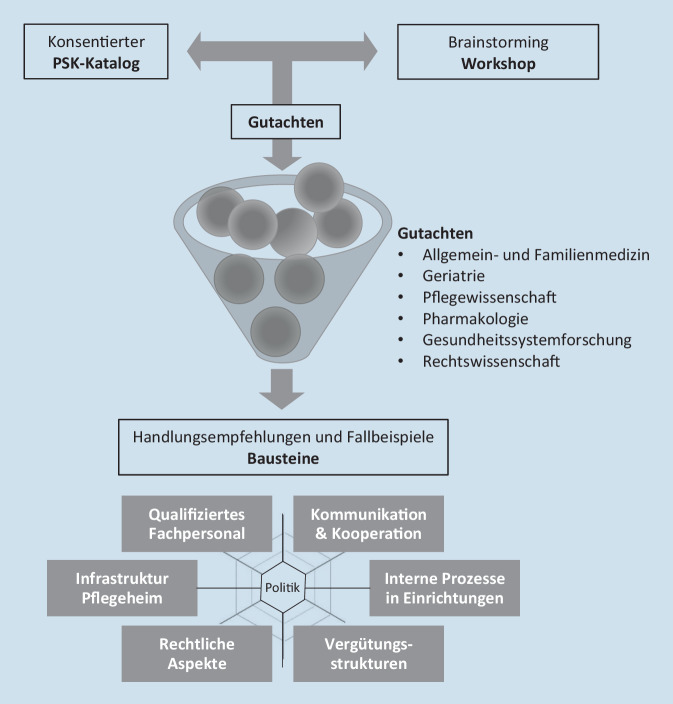

Die genannten Präventionspotenziale wurden unter der Annahme optimaler Versorgungsbedingungen eingeschätzt, die momentan nur selten gegeben sind. Deswegen wurden im Workshop-Brainstorming und mittels der sechs Gutachten mögliche Maßnahmen und Interventionen ausgearbeitet (s. Abb. 2). Die Stärkung der ambulanten Versorgung ist eine wesentliche Voraussetzung bei der Gestaltung einer guten Versorgungssituation. Die Empfehlungen der Expertinnen und Experten zur Vermeidung von Hospitalisierungen und damit zur Verbesserung der Versorgung von PHB wurden sechs Bausteinen einer guten Versorgungssituation zugeordnet. Die Politik, die richtungsweisend mittels Gesetzgebung, Empfehlungen und finanzieller Anreize eingreifen und intervenieren kann, steht dabei im Zentrum. Die sechs Bausteine sind im Einzelnen:

#### Baustein 1: *Qualifiziertes Fachpersonal*.

Die an der Versorgung beteiligten Fachpersonen müssen über die entsprechenden (Zusatz‑) Qualifikationen und Kompetenzen verfügen.

#### Baustein 2: *Kooperation & Kommunikation*.

Es müssen Kooperationsstrukturen geschaffen werden, die eine gelingende Zusammenarbeit ermöglichen, und es müssen transparente Kommunikations- und Informationswege vorhanden sein.

#### Baustein 3: *Infrastruktur*.

Es müssen räumliche, digitale und medizintechnische Voraussetzungen in den Pflegeeinrichtungen geschaffen werden, die eine Diagnostik und Behandlung vor Ort ermöglichen sowie die Kommunikation innerhalb des Netzwerks unterstützen.

#### Baustein 4: *Interne Prozesse in den Einrichtungen*.

Die Prozesse innerhalb der Einrichtungen sowie an den Schnittstellen zu den Kooperationspartnern müssen so gestaltet sein, dass sie eine gelingende Zusammenarbeit ermöglichen.

#### Baustein 5: *Rechtliche Rahmenbedingungen*.

Zur Weiterentwicklung der Versorgung müssen die gesetzlichen Rahmenbedingungen beachtet und/oder angepasst werden.

#### Baustein 6: *Vergütungsstrukturen.*

Für das Gelingen einer bedarfsgerechten Versorgung von PHB müssen bestehende Vergütungsstrukturen angepasst und weiterentwickelt werden.

Die Entwicklung der Handlungsempfehlungen mit Zuordnung zu Bausteinen ist schematisch in Abb. 2 dargestellt. Tab. 2 zeigt die Quintessenz der Handlungsempfehlungen für Berufsgruppen, Einrichtungen und weitere beteiligte Sektoren der PHB-zentrierten Versorgung. Die detaillierte Baustein-spezifische Darstellung der Handlungsempfehlungen ist im Onlinematerial (Supplementär-Tab. 2) zu finden.

**Table 2:** 

**Handlungsempfehlungen für die Berufsgruppen** *(z.* *B. Pflegefachpersonal, ambulant und klinisch tätige Ärztinnen und Ärzte, Apothekerinnen und Apotheker, Therapeutinnen und Therapeuten, Rettungsdienstfachpersonal)*	Digitale Kooperation und Kommunikation zur Abklärung und Diagnosestellung (telemedizinisch, evtl. inklusive technologie-diagnostisch unterstützte Videokonferenzen)
	Interprofessionelle Beratung und (telemedizinische) Konsultation z. B. durch (Fach‑)Ärzteschaft, pharmakologische Beratung durch Apotheker/-innen, Medikationsreview
	Erweiterung des geriatrisch-gerontologischen Wissens und der Fähig‑/Fertigkeiten
	Sensibilisierung für PSK-relevante Risikokonstellationen
	Erweiterung von Kenntnissen zur Pharmakologie und zur geriatrischen Medikationsproblematik (PRISCUS-Liste)
	Anwendung von Delegationskonzepten (z. B. VerAH, AGnES, EVA usw.) im Rahmen ärztlicher Hausbesuche in Pflegeheimen
	Einsatz einer Bezugspflegeperson
	Biografiearbeit mit PHB
	Interprofessionelle Zusammenarbeit mit flachen Hierarchien und gegenseitigem Respekt
**Handlungsempfehlungen für Einrichtungen** *(z.* *B. Pflegeheime, Krankenhäuser, Einrichtungsträger)*	**Zur Gewährleistung der Infrastruktur in Pflegeheimen:**
	Einrichtung eines Medizinraums mit Labor oder Kooperation mit mobilem „Diagnostik“-Bus
	Anschaffung von Material, Medizintechnik (Sonografie, Röntgen, EKG, (digitales) Stethoskop) und Kommunikationstechnik
	Einrichtung eines Internetzugangs in Behandlungszimmern bzw. im Medizinraum
	Anwendung von Telemedizin zwecks Diagnostik und Monitoring (per Video/Audio-Konsultation, inkl. Medikationsplan, Early Warning Score)
	Nutzung einer elektronischen einrichtungsübergreifenden PHB-Krankenakte
	Therapieziele digital zugänglich machen für Berufsgruppen sowie PHB und ihre Angehörige
	**Einrichtungsübergreifende Handlungsempfehlungen:**
	Durchführung der – interprofessionellen – Schulungen, Team- und Notfalltrainingsszenarien durch zertifizierte Träger, Ärztekammern, Einrichtungen usw.
	Durchführung (ethischer) einrichtungsinterner und sektorenübergreifender Fallkonferenzen
	Prozessbegleitung für sowie Einrichtung von Fall‑/Blitzkonferenzen zum momentanen Gesundheitszustand der PHB, Planung der Versorgung am Lebensende bei Aufnahme des/der PHB im Pflegeheim (ACP), Standardvorgehensweisen (SOP), Präventionsmaßnahmen
	Etablierung eines standardisierten Aufnahme- und Überleitungsmanagement
	Erweiterung und Verbesserung der Kooperation mit anderen Berufsgruppen in der Gesundheitsversorgung
	Anwendung von Unterbringungskonzepten bei PSK-Risikokonstellationen (evtl. Krankenhausbetten im Pflegeheim mit Klinikkooperation)
**Handlungsempfehlungen für weitere beteiligte Sektoren** *(z.* *B. Politik, Gesellschaft, Forschung, Religion, Kultur)*	Schaffung von Vergütungsstrukturen (z. B. für Schulungskosten, Abschreibung baulicher und Geräte-Investitionen, Mehraufwand der Netzwerkarbeit und PSK-Assessments, Vermeidungsbudget)
	Erstellung regionaler Rahmenvereinbarungen zu einer gemeinsamen Vision der Leistungserbringenden
	Entwicklung von Versorgungspfaden, inkl. Evaluationskonzept und Ergebniskontrolle, vor allem für Indikationen, die intensiven Austausch und interprofessionelle Beratung erfordern
	Erhöhung des Personalschlüssels insgesamt und speziell für qualifiziertes Pflegefachpersonal
	Sozialrechtliche Trennung von Behandlung, Rehabilitation und Pflege durch partielle Integration der Pflegeversicherung in die Krankenversicherung
	Anpassung der Heilkundeübertragungsrichtlinie hinsichtlich Stärkung der pflegefachlichen Kompetenz
	Einhaltung der Fort- und Weiterbildungsverpflichtung
	Durchführung von SGB-XI-finanzierten wissenschaftlichen Evaluationsstudien (Kosten-Nutzen-Analysen, Qualitative Befragungen von Berufsgruppen, PHB und Angehörigen, Monitoring der Hospitalisierungszahlen)

*ACP* Vorausplanung der Versorgung am Lebensende (Advanced Care Planning), *AGnES* Arztentlastende, Gemeindenahe, E‑Health-gestützte, Systemische Intervention durch qualifizierte Hausarztpraxismitarbeiter, *EKG* Elektrokardiogramm, *EVA* Entlastende Versorgungsassistent/-in (auch nicht-ärztliche Praxisassistent/-in genannt), *PHB* Pflegeheimbewohnerinnen und -bewohner, *PRISCUS* Liste mit potenziell inadäquater Medikation im Alter (Personen ab 65 Jahre), *PSK* Pflegeheim-sensitiver Krankenhausfall, *SGB XI* Elftes Buch Sozialgesetzbuch, *SOP* Standardvorgehensweise (Standard Operating Procedure), *VerAH* Versorgungsassistent/-in in der Hausarztpraxis

Die Wirkung von Handlungsempfehlungen ist in der Regel nicht auf eine PSK beschränkt, sondern mit jeder Maßnahme kann mehreren unterschiedlich begründeten Krankenhauseinweisungen vorgebeugt werden. Auch besteht die Möglichkeit der Kombination von Handlungsempfehlungen mit wahrscheinlich synergistischer Wirkung. Die Handlungsempfehlungen hängen untereinander eng zusammen: Änderungen in einem Bereich werden Folgen für andere Bereiche oder Sektoren haben. Deutlich wird, dass der Kommunikation und Kooperation zwischen allen Akteuren eine Schlüsselrolle zukommt, unterstützt wird diese durch eine erweiterte Fachkompetenz aller an der Versorgung beteiligten Berufsgruppen, durch die Einrichtungsinfrastruktur und die Standardisierung von Prozessabläufen.

## Diskussion

Die durchschnittlichen Hospitalisierungskosten der PHB unserer Stichprobe (€ 4030) liegen im Rahmen anderer Studien [45]. Die Krankenhausfälle mit einem Häufigkeitsanteil ≥ 0,1 % unter PHB wurden von einem Expertenpanel auf ihr Vermeidungspotenzial eingeschätzt. Im Falle statistisch signifikant unterschiedlich eingeschätzter Vermeidungspotenziale durch die vier Fachbereiche, die im Expertenpanel vertreten waren, stellte sich der Unterschied als sehr gering dar. Klinikerinnen und Kliniker waren etwas vorsichtiger mit der Schätzung der Vermeidbarkeitsanteile von Hospitalisierungen unter PHB. Dies dürfte darin begründet liegen, dass sie im Krankenhaus vor allem schwerer erkrankte Personen versorgen. 58 Pflegeheim-sensitive Krankenhausfälle (PSK) wurden konsentiert. Wir konnten, wie vermutet [20, 21], zeigen, dass das ambulante mit dem Pflegeheimsetting nicht vergleichbar ist. Die intensive 24/7-Versorgung sowie spezifische Merkmale des Pflegeheimklientels (Erkrankungskategorien, Multimorbidität, höheres Alter) ändern die Art und den Anteil der – potenziell vermeidbaren – Krankenhausfälle. Dies bestätigt den Bedarf an einem expliziten PSK-Katalog [6].

Mittels einer strukturierten und systematischen Vorgehensweise wurden Handlungsempfehlungen ausgearbeitet, die Ergebnisse aus der Schweiz und den USA [9, 46] bestätigen und erweitern.

Eine Maßnahme zur Erhöhung der geriatrisch-gerontologischen Fachkompetenz der beteiligten Berufsgruppen würde die Wachsamkeit gegenüber Frühwarnzeichen für eine Verschlechterung des Gesundheitszustandes der PHB erhöhen und eine Sensibilisierung für PSK und diesbezügliche Risikokonstellationen unterstützen.

Schwierigkeiten bei der Implementierung von Maßnahmen zeigt eine aktuelle Studie aus Deutschland: Hier wurden Ärztinnen und Ärzte, Pflegefachpersonal und Apothekerinnen und Apotheker zur Erhöhung der Arzneimitteltherapiesicherheit geschult. Eine Reduzierung von Krankenhauseinweisungen konnte nicht verzeichnet werden. Die Forscher berichteten u. a. von geringer Compliance der Ärzteschaft, die wiederum auf Systembedingungen (z. B. Ärztemangel im ambulanten Bereich) zurückgeführt werden kann [37].

Zu den zeitnah umzusetzenden Handlungsempfehlungen gehören die berufsgruppenübergreifende Kommunikation, Kooperation und Beratung, die zu einer verbesserten Patientensicherheit führen [47] und eine Reduktion der Hospitalisierungen aus dem Pflegeheim erwarten lassen [27, 32, 34]. Eine erste Inventarisierung von Kooperationen zwischen Ärzteschaft und Pflegefachpersonal in Deutschland lieferte mögliche Ansatzpunkte für Verbesserungen [29]. Diese wurden in dem Projekt „CoCARE“ mittels regelmäßiger ärztlicher Visiten, einer gemeinsamen Patientenakte und Medikamentenkontrollen adressiert [30]. International empfiehlt die Weltgesundheitsorganisation (WHO) für eine fokussierte Kommunikation zwischen ärztlich und pflegerisch tätigen Personen im Gesundheitswesen das „Situation-Background-Assessment-Recommendation“ (SBAR)-Tool^2^ [48–50]. Den PSK-Empfehlungen folgend, würde eine Bezugspflegeperson die Kommunikation zu Ärztinnen und Ärzten zusätzlich erleichtern. Dies wird für Deutschland auch durch regionale Versorgerteams dargelegt, in der allgemeinmedizinische bzw. fachärztliche Ressourcen gebündelt werden. Eine geschulte Pflegefachperson in der Pflegeinrichtung ist dabei Ansprechpartner für die Ärzteschaft [34].

Ferner sind als kurzfristige Maßnahmen eine Standardisierung von Prozessen hinsichtlich „Advanced Care Planning“ (ACP), ein Aufnahme- und Überleitungsmanagement und eine Videokonsultation per Telemedizin (auch bei basaler technischer Ausstattung bzw. ohne aufwändige Änderungen der finanziellen und rechtlichen Rahmenbedingungen) relativ schnell und einfach umzusetzen. Erste Interventionen dahingehend werden für das deutsche Gesundheitssystem aktuell erforscht (ACP [26], Telemedizin [36]).

Wie bei Givens et al. [51] wurde neurologischen und infektiösen Erkrankungen das höchste Vermeidungspotenzial zugeschrieben. Eine Hochrechnung auf Grundlage der Daten unserer Studie [6] deutet darauf hin, dass nach erfolgreicher Implementierung aller Handlungsempfehlungen insgesamt fast 35 % der Krankenhausaufenthalte unter Pflegeheimbewohnenden vermieden werden könnten.

Pneumonien und Frakturen wurden trotz hohen Fallaufkommens nicht in den PSK-Katalog aufgenommen, da sie die Kriterien für PSK mit eingeschätzten Vermeidungspotentialen unter 70 % nicht erfüllten, dennoch könnte der Einsatz mobiler Röntgengeräte viele dieser Hospitalisierungen vermeiden und eine leitliniengerechte Versorgung gewährleisten [52–54]. Nicht zuletzt könnten Maßnahmen der Primärprävention, die in unserer Studie bei der Einschätzung des Vermeidungspotenzials nicht berücksichtigt wurden, bei Pneumonien, Frakturen und Sepsis (vgl. Tab. 1) vorbeugend wirken und zur Vermeidung weiterer Einweisungen beitragen.

**Figure 3:**
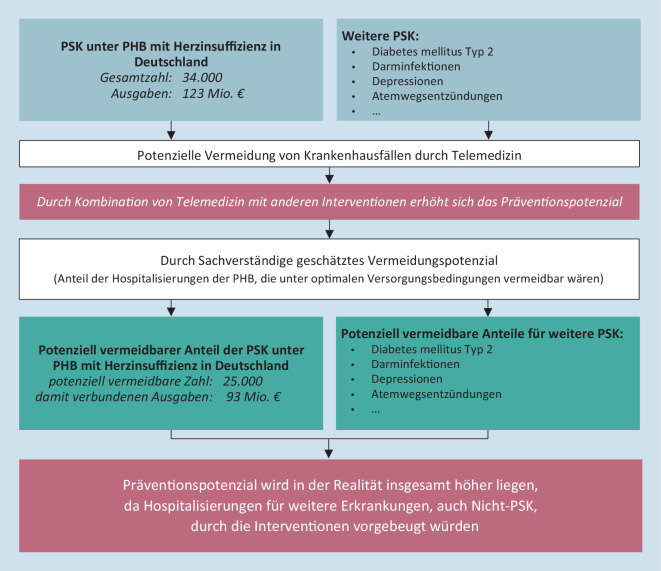

Unsere Ergebnisse zeigen auf, dass, mit der Umsetzung einer einzelnen Handlungsempfehlung mehreren PSK und darüber hinaus weiteren Krankenhausfällen – etwa aus den 117 häufigsten Einweisungsdiagnosen der Sekundärdatenanalyse – vorgebeugt werden kann. In Abb. 3 sind diese übergreifende potenzielle Relevanz und Wirksamkeit für den Einsatz der Telemedizin bei Herzinsuffizienz und weiteren Erkrankungen sowie die Relevanz der Kombination von Interventionen in den beiden rot hinterlegten Bereichen dargestellt. Telemedizinische Konsultationsmöglichkeiten können vertraglich geregelte Kooperationen von Pflegeheimen mit geriatrisch-gerontologisch qualifizierten ärztlich und therapeutisch tätigen Personen unterstützen. Eine Infrastruktur mit direktem Zugang zu Laboranalysen, EKG-Gerät, mobilem Röntgen, telemedizinisch integriertem Stethoskop und anderen Techniken kann die Diagnosestellung vor Ort für sowohl herzinsuffiziente als auch weitere erkrankte PHB unterstützen. Dazu soll Pflegefachpersonal für heilkundliche Tätigkeiten qualifiziert und befugt werden. Alle an der Versorgung der PHB Beteiligten sollten geriatrische bzw. gerontologische sowie PSK-relevante Risikokonstellationen erkennen und adäquat (be-)handeln können. Interdisziplinär entwickelte Behandlungspfade für PHB und die Dokumentation in einer für alle relevanten Stakeholder zugänglichen elektronischen Patientenakte können die Transparenz von Information und Kommunikation weiter erhöhen und z. B. ein regelmäßiges Update von Medikationsplänen ermöglichen.

Im internationalen Vergleich zeigte die Implementierung von Einzelmaßnahmen, wie die Einführung eines mobilen Röntgengeräts [55] oder eines Pflegeheim-Behandlungspfads für Pneumonie [53], eine 6 %- bzw. 12 %ige Reduktion der Hospitalisierungen unter PHB. Nationale Initiativen konnten in den USA erfolgreich die Einweisungen unter PHB für mehrere chronische Erkrankungen wie Demenz, Herzinsuffizienz und chronisch obstruktive Lungenerkrankung (COPD) um jeweils ca. 9 % reduzieren [56]. Besonders gute Erfolge wurden mit Kombinationen von Interventionen erzielt: Bezugspflege zusammen mit Kooperationsmanagement und einrichtungsbezogenen Standardvorgehensweisen (SOPs), wie etwa der Etablierung eines initialen Risiko-Assessments und eines Überleitungsmanagements, reduzierten die Hospitalisierungen unter PHB um 40 % [57]. Durch den kombinierten Einsatz einer/eines Advanced Practice Registered Nurse (APN), eines Unterstützungsteams aus Sozialarbeiterinnen und Sozialarbeitern sowie Coaches zur Qualitätsverbesserung konnte zusammen mit Feedback zu (potenziell vermeidbaren) Krankenhausfällen und Gesundheitsinformationstechnologie die Hospitalisierung unter PHB um 30 % reduziert werden [58]. Diese Ergebnisse unterstützen die auf Basis unserer Studienergebnisse erwartete Reduktion der Hospitalisierungen unter PHB in Deutschland von bis zu 35 %.

Basierend auf dem PSK-Katalog werden konkrete Ansatzpunkte zur Verbesserung einer personenzentrierten Versorgung und Erhöhung der Patientensicherheit sowie Möglichkeiten der Refinanzierung aufgezeigt. Somit bietet der PSK-Katalog einen relevanten Beitrag für die (Re-)Organisation des Gesundheitswesens.

### Limitationen

Die Sekundärdatenanalyse beruht auf Krankenhausentlassungsdiagnosen, die nicht zwangsläufig den Einweisungsgrund betrafen. Der von den Expertinnen und Experten geforderte erfahrungsbasierte Rückschluss auf den Anlass der Hospitalisierung stellt eine Limitation der gewählten Vorgehensweise dar. Bereits bei der Planung der Studie wurde der Vorbereitung des Expertenpanels besondere Aufmerksamkeit gewidmet. Trotz dieser Bemühungen ist nicht auszuschließen, dass es zu Fehleinschätzungen gekommen ist, die eine Über- oder eine Unterschätzung des Vermeidungspotenzials zur Folge hätten. Eine weitere Limitation liegt darin, dass Ergebnisse für geplante und ungeplante Krankenhausaufenthalte nicht differenziert ausgewiesen werden konnten, da wir diese nicht getrennt erhoben haben. Durch Hinweise an das Expertenpanel wurde der Anteil der vermeidbaren Einweisungen vor allem auf ungeplante Einweisungen ausgerichtet, die für die Pflegepraxis und ggf. auch für die Beurteilung der Qualität der stationären Versorgung Pflegebedürftiger am interessantesten sein dürften.

Die Multidisziplinarität und die ausgewiesene Erfahrung des Expertenpanels, die geringen Unterschiede zwischen den Fachbereichen bei der Einschätzung des Vermeidungspotenzials [6], die im Vergleich zu anderen Delphi-Verfahren relativ hohe Zahl an inkludierten Expertinnen und Experten und die sehr hohe Responserate von 91 % machen es eher unwahrscheinlich, dass ein anderes Panel zu anderen Ergebnissen gekommen wäre. Die Berücksichtigung der „optimalen Versorgungsbedingungen“, zum Beispiel ein Röntgenbefund durch Einsatz eines mobilen Röntgengeräts bei der Abstrahierung der Vermeidungspotenziale für Pneumonien und Frakturen, fiel den Expertinnen und Experten in den Delphi-Runden nicht immer leicht. Die konsentierten Vermeidungspotenziale dürften tendenziell Untergrenzen darstellen, da sie basierend auf dem Wissen um die jetzigen – nicht immer optimalen – Versorgungsbedingungen wahrscheinlich eher restriktiv eingeschätzt wurden.

Der Horizont der Wirksamkeit einzelner Handlungsempfehlungen konnte aufgrund vieler Abhängigkeiten zwischen den Interventionen nur grob geschätzt werden. Bei der einrichtungsbezogenen Priorisierung einzelner Maßnahmen kann er, unter Berücksichtigung finanzieller Aspekte, jedoch der Orientierung dienen.

## Fazit

Verschiedene an der Versorgung von Pflegeheimbewohnerinnen und -bewohnern (PHB) Beteiligte können durch die Implementierung erster Handlungsempfehlungen zur Vermeidung von Hospitalisierungen vor allem bei neurologischen und infektiösen Erkrankungen beitragen. Der konsensvalidierte PSK-Katalog aus der zweiten Phase des PSK-Projektes deckt Fälle mit besonders hohem Vermeidungspotenzial auf. Dadurch sensibilisiert er für PSK (Pflegeheim-sensitive Krankenhausfälle) und motiviert die Beteiligten für die Umsetzung der in diesem Aufsatz erwähnten Maßnahmen. Handlungsempfehlungen zur Reduzierung von Hospitalisierungen von Pflegeheimbewohnerinnen und -bewohnern sind vielumfassend und betreffen die sechs miteinander verbundenen Bausteine der Kommunikation, Kooperation, Dokumentation und Versorgungskompetenz sowie einrichtungsbezogene finanzielle und rechtliche Aspekte. Mit einer effektiven Kombination und einer sektorenübergreifenden Implementierung der vorliegenden Handlungsempfehlungen könnte fast 35 % aller Hospitalisierungen (für Deutschland fast 220.000 Krankenhausfälle) von Pflegeheimbewohnerinnen und -bewohnern vorgebeugt werden. Insgesamt sind diese vermeidbaren Krankenhausbehandlungen mit Ausgaben in Höhe von rund 768 Mio. € verbunden. Während die Umsetzung einzelner Maßnahmen bereits Wirkung zeigt, verspricht die Kombination mehrerer Handlungsempfehlungen besonderes Potenzial. Für einen wirksamen Transfer der Handlungsempfehlungen in die Praxis ist eine Unterstützung durch die (Gesundheits‑)Politik erforderlich. Viele innovative Ansätze können zunächst in Modellprojekten erprobt, optimiert und dann breit implementiert werden. Es wird einer gemeinsamen Anstrengung aller Beteiligten inklusive gesundheitspolitischer Unterstützung sowie weitreichender Änderungen der bestehenden Versorgung von Pflegeheimbewohnerinnen und -bewohnern bedürfen, um das Vermeidungspotenzial von 35 % (220.000 Krankenhauseinweisungen pro Jahr) zu heben und damit die Lebensqualität der hochbetagten Pflegebedürftigen zu verbessern.

## Supplementary Information





## Footnotes

1 https://innovationsfonds.g-ba.de/innovationsausschuss/; Zugriffsdatum: 19.10.2022.

2 https://www.ihi.org/resources/Pages/Tools/SBARToolkit.aspx; Zugriffsdatum: 19.10.2022.
