# Supplementary material for: Nursing home-sensitive conditions and approaches to reduce hospitalization of nursing home residents
Source: Bundesgesundheitsblatt Gesundheitsforschung Gesundheitsschutz. 2023 Jan 10;66(2):199–211. [Article in German] doi: 10.1007/s00103-022-03654-4 (PMC9830609; doi:10.1007/s00103-022-03654-4)
Supplement: Supplementary file 1 [file 103_2022_3654_MOESM1_ESM.pdf]

Supplement zur Originalarbeit:

Pflegeheim-sensitive Krankenhausfälle und Ansätze zur Verringerung der Hospitalisierung von Pflegeheimbewohnerinnen und -bewohnern

Autoren: Maria Paula Valk-Draad[1,2], Sabine Bohnet-Joschko[1], PSK-Studiengruppe

Collaborators: PSK-Studiengruppe: Maria Paula Valk-Draad[1,2], Katja Stahl[3,4], Christel Bienstein[5], Hans-Jürgen Heppner[6,7], Andreas Sönnichsen[8], Hagen Sjard Bachmann[9], Petra Thuermann[10,11], Oliver Gröne[3], Paula Zietzsch[3], Helmut Hildebrandt[3], Thomas Klie[12], Sabine Bohnet-Joschko[1]

Afilliations:

1. Lehrstuhl für Management und Innovation im Gesundheitswesen, Fakultät für Wirtschaft und Gesellschaft, Universität Witten/Herdecke, Witten, Deutschland
2. Lehrstuhl für Community Health Nursing, Fakultät für Gesundheit, Universität Witten/Herdecke, Witten, Deutschland
3. OptiMedis AG, Hamburg, Deutschland
4. Institut für Gesundheitswissenschaft, Universität zu Lübeck, Lübeck, Deutschland
5. Pflege e. V., Berlin, Deutschland
6. Lehrstuhl für Geriatrie, Fakultät für Gesundheit, Universität Witten/Herdecke, Witten, Deutschland
7. Lehrstuhl für Geriatrie, Institut für Biomedizin des Alterns, FAU Erlangen-Nürnberg, Erlangen, Deutschland
8. Facharztpraxis für Innere Medizin und Allgemeinmedizin, Salzburg, Österreich
9. Lehrstuhl für Pharmakologie und Toxikologie, Fakultät für Gesundheit, Universität Witten/Herdecke, Witten, Deutschland
10. Lehrstuhl für Klinische Pharmakologie, Fakultät für Gesundheit, Universität Witten/Herdecke, Witten, Deutschland
11. Philipp Klee-Institut für Klinische Pharmakologie am Helios Universitätsklinikum Wuppertal, Wuppertal, Deutschland
12. AGP Sozialforschung, FIVE – Forschungs- und Innovationsverbund der Evangelischen Hochschule Freiburg e.V., Freiburg, Deutschland

Copyright: License: Creative Commons CC-BY Attribution 4.0 International. Credit must be given to the creators

Inhalt

Hinweise zur Einschätzung des Vermeidungspotenzials an das Expertenpanel 2
Supplementär-Tabelle 1 4
Supplementär-Tabelle 2 7

Dieses Projekt wurde mit Mitteln des Innovationsausschusses beim Gemeinsamen Bundesausschuss unter dem Förderkennzeichen 01VSF18026 gefördert.

Hinweise zur Einschätzung des Vermeidungspotenzials an das Expertenpanel

Nachfolgend eine Zusammenfassung unserer Hinweise an das Expertenpanel:

Vollständige Hinweise, in den Extended Data zu Referenz 6 im Manuskript:

> *Referenz 6 im Manuskript: Bohnet-Joschko S, Valk-Draad M, Schulte T, Groene O (2022) Nursing home-sensitive conditions: analysis of routine health insurance data and modified Delphi analysis of potentially avoidable hospitalizations [version 2; peer review: 2 approved]. F1000Research 10:1223. https://doi.org/10.12688/f1000research.73875.2;*
>
> *Mit Extended Data unter: https://osf.io/eaj58*

Im ersten Fragebogen:

1. Die ICD-10-Codes betreffen Entlassungsdiagnosen, wohlwissend, dass diese weniger die Einweisungsdiagnose widerspiegeln, sondern eher eine zugrundeliegende Erkrankung, die vielleicht auch für die Abrechnung mittels DRGs vorteilhaft sein mag. Stellen Sie sich daher bei der Einschätzung bitte vor, welche tatsächliche Einweisungsdiagnose der angegebenen Entlassungsdiagnose in der Versorgungsrealität am ehesten zugrunde liegt und abstrahieren Sie bitte, bei wie viel Prozent der jeweiligen Patienten eine Krankenhauseinweisung ggf. vermeidbar wäre, unter Berücksichtigung des Einflusses von Komorbidität sowie Schwere der Erkrankung.
2. Bitte beziehen Sie in Ihrer Einschätzung die Existenz eventueller Patientenverfügungen und Präferenzen hinsichtlich der Behandlung von Angehörigen nicht mit ein, auch wenn sie für die Pflegeheimbewohnenden in der Praxis noch so relevant sind.
3. Grundannahmen zu optimalen Versorgungsbedingungen:
   a) gut geschultes Pflegefachpersonal (kann Infusionen anlegen, kann Katheter wechseln, verfügt über grundlegende Kenntnisse der Diagnosestellung bzgl. der Notwendigkeit einer Krankenhauseinweisung, kann den Fall mit einem Arzt zielgerichtet besprechen),
   b) die Einrichtung hat die benötigten Ressourcen (z.B. für eine vorläufige Untersuchung, die Überwachung einer möglichen Krise im Zustand des Pflegeheimbewohnenden und die Einleitung von Behandlungsmaßnahmen, wie die Verwendung von hochwertigem Verbandsmaterial, das Anlegen von Infusionen und das Wechseln von Kathetern),
   c) gute Kooperations- und Rücksprachemöglichkeiten mit der ambulanten und klinischen ärztlichen Versorgung (z.B. mit einem Arzt/einer Ärztin der Allgemein-/ Familienmedizin, einem Facharzt/einer Fachärztin, z. B. der Neurologie, Geriatrie, Orthopädie, Kardiologie).
4. Ambulante Termine und palliative Termine werden nicht als Krankenhauseinweisung betrachtet.

Im zweiten Fragebogen:

Wenn Ihre Einschätzung abhängig ist von bestimmten Merkmalen:

- das Ausmaß der Symptome,
- das Vorhandensein von weiteren Erkrankungen,
- die Schwere der Erkrankung,
- die Notwendigkeit einer OP,
- die palliative Versorgung des Pflegeheimbewohnenden.

<u>*Unsere Empfehlung*</u>

- Fragen Sie sich, welche Fälle Sie überwiegend versorgen und wie viele davon im Pflegeheim behandelt werden können. Schätzen Sie das Vermeidungspotenzial dann bitte anteilmäßig ein. Diesen Endwert geben Sie dann bitte an.
- Ambulante Diagnostik und Erst-/Akutversorgung sowie Bewohner-/Angehörigenwünsche dabei bitte NICHT mit einbeziehen (siehe auch die nachfolgenden beiden Empfehlungen).

Wenn Ihre Einschätzung abhängig ist von der Dauer der Erkrankung, ob die Erkrankung akut aufgetreten ist, oder erst jetzt symptomatisch wird.

<u>*Unsere Empfehlung*</u>

- Im Falle von Erst- oder Folgediagnostik bzw. Akutversorgung oder therapeutischer Einstellung ist die Krankenhausbehandlung oft ambulant möglich. Ambulante Diagnostik und Behandlungen zählen nicht als Krankenhauseinweisung. Schätzen Sie das Vermeidungspotenzial bitte nur für die stationäre Behandlung ein und gehen Sie von optimalen Bedingungen im Pflegeheim aus, die weiter unten ausführlich beschrieben werden.

Wenn Ihre Einschätzung abhängig ist von den Wünschen der Pflegeheimbewohnenden und/oder ihren Angehörigen.

<u>*Unsere Empfehlung*</u>

- Beziehen Sie diese bitte nicht in Ihre Einschätzung mit ein: Wir möchten für diese Befragung lediglich wissen, was medizinisch unter optimalen Bedingungen möglich ist.

Supplementär-Tabelle 1. Präventionspotenzial der Pflegeheim-sensitiven Krankenhausfälle, rechts in der Tabelle absteigend sortiert, aufgrund von Fallaufkommen, Vermeidungspotenzial, sowie durchschnittlichen Krankenhauskosten pro Fall.

Nr.	ICD[a]	ICD-Kurzbeschreibung	Anteil der potenziell vermeidbaren Krankenhausfälle (%)[b]	Fälle gesamt hochgerechnet für Deutschland	Vermiedene Fälle hochgerechnet für Deutschland	Fälle-Anteil an allen Krankenhausfälle unter PHB[c]	Krankenhauskosten pro Fall	Hochrechnung Gesamtkosten je ICD in Deutschland	Präventionspotenzial: Hochrechnung vermiedener Gesamtkosten je PSK[d] in Deutschland
1	I50	Herzinsuffizienz	75	33.524	25.143	5,19%	3.682,9 €	123.465.251 €	92.598.939 €
2	I70	Atherosklerose	80	8.401	6.721	1,30%	6.912,4 €	58.072.590 €	46.458.072 €
3	N39	Sonstige Krankheiten des Harnsystems	85	20.775	17.659	3,22%	2.612,6 €	54.277.309 €	46.135.713 €
4	J44	Sonstige chronische obstruktive Lungenkrankheit	75	13.935	10.451	2,16%	4.345,7 €	60.555.145 €	45.416.359 €
5	G40	Epilepsie	90	14.016	12.614	2,17%	3.371,4 €	47.252.210 €	42.526.989 €
6	E86	Volumenmangel	75	22.224	16.668	3,44%	2.548,7 €	56.642.242 €	42.481.682 €
7	E11	Diabetes mellitus, Typ 2	90	10.719	9.647	1,66%	4.197,3 €	44.989.542 €	40.490.588 €
8	F20	Schizophrenie	75	5.712	4.284	0,88%	8.143,6 €	46.518.501 €	34.888.876 €
9	F05	Delir, nicht durch Alkohol oder andere psychotrope Substanzen bedingt	75	6.888	5.166	1,07%	5.914,9 €	40.740.774 €	30.555.580 €
10	L89	Dekubitalgeschwür und Druckzone	85	3.523	2.995	0,55%	8.282,0 €	29.180.119 €	24.803.101 €
11	J20	Akute Bronchitis	90	8.658	7.792	1,34%	2.692,3 €	23.310.093 €	20.979.084 €
12	F06	Andere psychische Störungen aufgrund einer Schädigung oder Funktionsstörung des Gehirns oder einer körperlichen Krankheit	75	3.273	2.455	0,51%	6.952,9 €	22.759.278 €	17.069.459 €
13	N30	Zystitis	90	6.192	5.573	0,96%	2.770,7 €	17.156.073 €	15.440.466 €
14	G30	Alzheimer-Krankheit	90	2.753	2.478	0,43%	5.988,9 €	16.488.068 €	14.839.261 €
15	G20	Primäres Parkinson-Syndrom	80	3.216	2.573	0,50%	5.167,1 €	16.617.039 €	13.293.631 €
16	F33	Rezidivierende depressive Störung	85	1.706	1.450	0,26%	8.730,5 €	14.893.613 €	12.659.571 €
17	A04	Sonstige bakterielle Darminfektionen	80	3.588	2.870	0,56%	3.950,2 €	14.171.324 €	11.337.059 €
18	J22	Akute Infektion der unteren Atemwege, nicht näher bezeichnet	80	4.729	3.783	0,73%	2.923,3 €	13.825.222 €	11.060.178 €
19	A09	Sonstige und nicht näher bezeichnete Gastroenteritis und Kolitis infektiösen und nicht näher bezeichneten Ursprungs	80	6.020	4.816	0,93%	2.210,2 €	13.304.599 €	10.643.679 €
20	A46	Erysipel [Wundrose]	80	4.577	3.662	0,71%	2.893,9 €	13.246.042 €	10.596.833 €

Nr.	ICD[a]	ICD-Kurzbeschreibung	Anteil der potenziell vermeid-baren Kranken-hausfälle (%)[b]	Fälle ge-samt hoch-gerechnet für Deutsch-land	Vermie-dene Fälle hochge-rechnet für Deutsch-land	Fälle-Anteil an allen Kran-kenhaus-fälle unter PHB[c]	Kranken-hauskos-ten pro Fall	Hochrechnung Ge-samtkosten je ICD in Deutschland	Präventionspoten-zial: Hochrech-nung vermiedener Gesamtkosten je PSK[d] in Deutsch-land
21	I10	Essentielle (primäre) Hypertonie	90	5.652	5.086	0,88%	2.068,7 €	11.691.200 €	10.522.080 €
22	D50	Eisenmangelanämie	80	3.915	3.132	0,61%	3.035,2 €	11.883.390 €	9.506.712 €
23	N18	Chronische Nierenkrankheit	85	2.412	2.050	0,37%	4.541,9 €	10.954.882 €	9.311.650 €
24	K59	Sonstige funktionelle Darmstörungen	90	5.358	4.822	0,83%	1.930,3 €	10.341.648 €	9.307.483 €
25	K21	Gastroösophageale Refluxkrankheit	90	4.486	4.037	0,69%	2.215,4 €	9.938.276 €	8.944.449 €
26	K57	Divertikulose des Darmes	75	2.854	2.141	0,44%	4.113,9 €	11.743.012 €	8.807.259 €
27	K29	Gastritis und Duodenitis	80	4.456	3.565	0,69%	2.314,4 €	10.312.005 €	8.249.604 €
28	S00	Oberflächliche Verletzung des Kopfes	90	7.979	7.181	1,24%	1.121,8 €	8.950.789 €	8.055.710 €
29	C44	Sonstige bösartige Neubildungen der Haut	75	2.952	2.214	0,46%	3.017,3 €	8.908.335 €	6.681.251 €
30	F01	Vaskuläre Demenz	90	1.355	1.219	0,21%	5.351,7 €	7.249.392 €	6.524.453 €
31	F07	Persönlichkeits- und Verhaltensstörung aufgrund einer Krankheit, Schädigung oder Funktionsstörung des Gehirns	90	1.071	964	0,17%	6.327,0 €	6.775.288 €	6.097.759 €
32	K25	Ulcus ventriculi	75	1.703	1.277	0,26%	4.742,5 €	8.074.243 €	6.055.683 €
33	E87	Sonstige Störungen des Wasser- und Elektrolyt-haushaltes sowie des Säure-Basen-Gleichgewichts	70	2.895	2.027	0,45%	2.851,3 €	8.254.475 €	5.778.133 €
34	G35	Multiple Sklerose [Encephalomyelitis disseminata]	75	1.446	1.084	0,22%	5.180,8 €	7.490.409 €	5.617.807 €
35	R26	Störungen des Ganges und der Mobilität	85	1.395	1.186	0,22%	4.670,4 €	6.515.815 €	5.538.443 €
36	F10	Psychische und Verhaltensstörungen durch Alkohol	80	2.074	1.659	0,32%	3.334,4 €	6.915.927 €	5.532.742 €
37	D64	Sonstige Anämien	80	2.159	1.727	0,33%	2.809,8 €	6.065.254 €	4.852.203 €
38	I80	Thrombose, Phlebitis und Thrombophlebitis	75	2.861	2.146	0,44%	2.256,1 €	6.455.192 €	4.841.394 €
39	M54	Rückenschmerzen	85	1.946	1.654	0,30%	2.519,2 €	4.901.847 €	4.166.570 €
40	H25	Cataracta senilis	95	2.804	2.664	0,43%	1.516,6 €	4.252.292 €	4.039.678 €
41	K26	Ulcus duodeni	75	1.236	927	0,19%	4.267,8 €	5.276.529 €	3.957.397 €
42	K52	Sonstige nichtinfektiöse Gastroenteritis und Kolitis	75	2.027	1.520	0,31%	2.599,8 €	5.269.354 €	3.952.015 €
43	J10	Grippe durch saisonale nachgewiesene Influenzavi-ren	75	1.429	1.072	0,22%	3.669,5 €	5.243.469 €	3.932.602 €
44	A08	Virusbedingte und sonstige näher bezeichnete Dar-minfektionen	80	1.618	1.294	0,25%	2.687,3 €	4.348.381 €	3.478.705 €

Nr.	ICD[a]	ICD-Kurzbeschreibung	Anteil der potenziell vermeidbaren Krankenhausfälle (%)[b]	Fälle gesamt hochgerechnet für Deutschland	Vermiedene Fälle hochgerechnet für Deutschland	Fälle-Anteil an allen Krankenhausfälle unter PHB[c]	Krankenhauskosten pro Fall	Hochrechnung Gesamtkosten je ICD in Deutschland	Präventionspotenzial: Hochrechnung vermiedener Gesamtkosten je PSK[d] in Deutschland
45	R13	Dysphagie	80	1.746	1.397	0,27%	2.201,7 €	3.845.104 €	3.076.083 €
46	K62	Sonstige Krankheiten des Anus und des Rektums	75	1.253	940	0,19%	3.255,6 €	4.080.164 €	3.060.123 €
47	S70	Oberflächliche Verletzung der Hüfte und des Oberschenkels	85	2.206	1.875	0,34%	1.510,4 €	3.331.754 €	2.831.991 €
48	J40	Bronchitis, nicht als akut oder chronisch bezeichnet	90	1.233	1.110	0,19%	2.492,7 €	3.073.507 €	2.766.156 €
49	F32	Depressive Episode	85	611	520	0,09%	5.244,8 €	3.206.822 €	2.725.798 €
50	S30	Oberflächliche Verletzung des Abdomens, der Lumbosakralgegend und des Beckens	80	2.034	1.627	0,31%	1.462,5 €	2.974.189 €	2.379.351 €
51	I95	Hypotonie	85	1.544	1.312	0,24%	1.801,8 €	2.781.535 €	2.364.305 €
52	L02	Hautabszess, Furunkel und Karbunkel	100	726	726	0,11%	3.176,5 €	2.307.037 €	2.307.037 €
53	S80	Oberflächliche Verletzung des Unterschenkels	90	872	784	0,13%	2.913,8 €	2.539.470 €	2.285.523 €
54	R11	Übelkeit und Erbrechen	80	1.334	1.067	0,21%	1.786,1 €	2.383.226 €	1.906.581 €
55	R07	Hals- und Brustschmerzen	70	1.831	1.282	0,28%	1.259,7 €	2.306.441 €	1.614.509 €
56	K08	Sonstige Krankheiten der Zähne und des Zahnhalteapparates	85	662	563	0,10%	2.362,1 €	1.563.954 €	1.329.361 €
57	S20	Oberflächliche Verletzung des Thorax	90	635	572	0,10%	1.563,0 €	992.597 €	893.337 €
58	S01	Offene Wunde des Kopfes	75	976	732	0,15%	1.007,2 €	983.325 €	737.493 €
		**Gesamt**		**270.174**	[e,f]**219.955**	[g]**41,84%**		**951.645.564 €**	**768.304.547 €**

[a] ICD: 10. Edition der International Classification of Diseases, deutsche Version, Dreisteller (ICD-10-GM-Dreisteller)

[b] Medianer Anteil der potenziell vermeidbaren Krankenhausfälle unter Pflegeheimbewohnerinnen und –bewohnern, wie vom Sachverständigenpanel unter der Prämisse optimaler Versorgungsbedingungen eingeschätzt.

[c] PHB: Pflegeheimbewohnerinnen und -bewohner

[d] PSK: Pflegeheim-sensitiver Krankenhausfall

[e] Anteil der tatsächlich durch die Handlungsempfehlungen möglich zu vermeidenden Fälle an allen potenziell vermeidbaren Krankenhausfällen (PSK) beträgt 81% (219.955/270.174*100)

[f] Anteil der tatsächlich durch die Handlungsempfehlungen möglich zu vermeidenden Fälle an allen Krankenhausaufenthalte unter Pflegeheimbewohnenden beträgt 34% (219.955/645.798*100)

[g] Anteil aller PSK-Fälle an allen Krankenhausaufenthalte unter Pflegeheimbewohnenden beträgt 41,84% (270.174/645.798*100)

Supplementär-Tabelle 2. Übersicht der Handlungsempfehlungen inklusive Zeithorizont der Wirksamkeit

Bausteine und Zeit-horizont	Unterpunkte	Erläuterungen, auch zu Zeithorizont
**1. QUALIFIZIERTES FACHPERSONAL**	1.1 Schulung & Weiterbildung der Haus-/ Fachärzte und –ärztinnen zu PSK-relevanten Risikokonstellationen	1.1.1. Schulung und Weiterbildung von Haus- und ggf. auch Fachärzte und -ärztinnen mit Fokus auf geriatrische/gerontologische Versorgungsaspekte im Zusammenhang von Erkrankungen, die das Risiko für eine (vermeidbare) Krankenhauseinweisung maßgeblich erhöhen (PSK-relevanten Risikokonstellationen) sowie hinsichtlich der Besonderheiten der palliativmedizinischen Versorgung<br>1.1.2. Die Durchführung der Schulungen und Weiterbildungen kann beispielsweise über Ärztekammern, Krankenhäuser, Pflegeeinrichtungen oder auch andere zertifizierte Träger erfolgen
	1.2 Stärkung pflegefachlicher Kompetenz & selbstständige Ausübung heilkundlicher Tätigkeiten durch Pflegekräfte (z. B. Advanced Practice Nurse (APN))	1.2.1 Schulung von Pflegekräften in heilkundlichen und behandlungspflegerischen Fragen (Wundversorgung, Schmerzmanagement, gerontopsychiatrische und palliativmedizinische Grundkenntnisse, Prävention, Biografiearbeit etc.), auch unter Rückgriff auf die Fort- und Weiterbildungsverpflichtung der Einrichtungsträger<br>1.2.2 Gezielte Schulung und Weiterbildung der an der Versorgung der PHB beteiligten Pflegekräfte zu Erkrankungen, die das Risiko für eine (vermeidbare) Krankenhauseinweisung maßgeblich erhöhen (PSK-relevante Risikokonstellationen) (Symptomeinschätzung, Verdachtsdiagnose) , auch unter Rückgriff auf die Fort- und Weiterbildungsverpflichtung der Einrichtungsträger<br>1.2.3 Anpassung der Ausbildungscurricula an die tatsächlichen Tätigkeitsanforderungen (Erweiterung des Spektrums medizinisch-pflegerischer Tätigkeiten, Kompetenzen für Assessment und Diagnostik, pharmakologische Grundkenntnisse) zur Erleichterung der Delegation ärztlicher Tätigkeiten und Stärkung der interprofessionellen Zusammenarbeit<br>1.2.4 Prüfung der Effektivität von Delegationskonzepten (z. B. VerAH, AGNES, EVA etc.) im Rahmen ärztlicher Hausbesuche in Pflegeheimen
	1.3 Schulung & Weiterbildung weiterer Berufsgruppen zu PSK-relevanten Risikokonstellationen	1.3.1 Strukturierte curriculare Fortbildung von Apotheker/ -innen zur klinischen Pharmazie bei PHB verbunden mit einem Ausbau der Beratungs- und Medikationsreview-Aufgaben der Apotheker/ -innen<br>1.3.2 Gezielte Schulung und Weiterbildung der an der Versorgung der PHB beteiligten Therapeuten zu Erkrankungen, die das Risiko für eine (vermeidbare) Krankenhauseinweisung maßgeblich erhöhen (PSK-relevante Risikokonstellationen)<br>1.3.3 Schulung von Mitarbeitenden des Rettungsdienstes, um im Einzelfall kriterienbasiert (Early Warning Score) und rechtssicher entscheiden zu können, ob PHB nicht in ein Krankenhaus transportiert, sondern an den ambulanten ärztlichen Versorgungsbereich überwiesen werden können
	1.4 Sektorenübergreifende, interprofessionelle Fortbildungen	1.4.1 Etablierung von regelmäßigen, interprofessionellen Fortbildungen der an der Versorgung der PHB beteiligten Berufsgruppen zu Erkrankungen, die das Risiko für eine (vermeidbare) Krankenhauseinweisung maßgeblich erhöhen (PSK-relevante Risikokonstellationen)<br>1.4.2 Weiterentwicklung bestehender Team- und Notfalltrainingsszenarien auf PSK-relevante Situationen in stationären Pflegeeinrichtungen
Zeithorizont:<br>*Kurz bis mittelfristig umsetzbar*		*Zu Zeithorizont: In Abhängigkeit von der Verfügbarkeit von inhaltlich ausgestalteten Konzepten kann die Einrichtung des Schulungs- und Weiterbildungsangebots sowohl für Ärzteschaft, Pflegefachpersonal und Therapeuten kurz- bis mittelfristig erfolgen. Für eine Anpassung der Ausbildungscurricula sowie deren Umsetzung in den bestehenden Ausbildungsstrukturen werden mehrere Jahre erforderlich sein. Interdisziplinäre Fortbildungen, Team- und Notfalltrainings können kurzfristig etabliert werden, sofern entsprechende Fortbildungs- und Simulationskonzepte verfügbar sind.*

Bausteine und Zeithorizont	Unterpunkte	Erläuterungen, auch zu Zeithorizont
**2. KOMMUNIKATION UND KOOPERATION**	2.1 Aufbau eines digital unterstützten, sektorenübergreifenden, alters- und bedarfsgerechten Versorgungsnetzwerks aus regionalen Leistungserbringenden	2.1.1 Abschluss von regionalen Rahmenvereinbarungen der Leistungserbringenden zur Zusammenarbeit (gemeinsame Vision) im Netzwerk<br>Dies kann beispielsweise im Sinne einer formalisierten Willensbekundung zu einer wertschätzenden und respektvollen Zusammenarbeit zwischen den Beteiligten (in einer Region) erfolgen. Die gemeinsame Vision für die Zusammenarbeit ist eine bedarfsgerechte und teilhabeorientierte Versorgung, unter weitestmöglicher Wahrung der Autonomie der PHB (Pflege-Charta), der Patientenrechte und Vermeidung von PSK. Eine solche Rahmenvereinbarung kann dazu beitragen, die Abhängigkeit der Kooperationsqualität von ökonomischen Handlungslogiken, intrinsischer Motivation, etc. zu verringern, da ein gemeinsames Ziel vereinbart wird, dem sich alle verpflichten.<br>2.1.2 Entwicklung von einrichtungsspezifischen, zuverlässigen Lösungen zur Kooperation mit niedergelassener Haus- und Fachärzteschaft, inkl. regelmäßiger Visiten (dabei muss den PHB freigestellt sein, von wem sie sich ärztlich versorgen lassen)<br>2.1.3 Sicherstellung der zuverlässigen Erreichbarkeit von Therapeuten (Physiotherapie, Ergotherapie, Logotherapie, etc.) durch einrichtungsspezifische Kooperationslösungen<br>2.1.4 Ausbau telemedizinischer Ansätze zur Abklärung und Diagnosestellung<br>2.1.5 Beitritt zu interprofessionellen Netzwerken (z. B. SAPV gem. §37b SGB V, ambulante Hospizdienste gem. §39a, Abs. 2, SGB V, Wundmanagement)
	2.2 Etablierung klar definierter, transparenter Kommunikations- und Informationswege	2.2.1 Einrichtung regelmäßiger, strukturierter Fallkonferenzen auf unterschiedlichen Ebenen (fach- und sektorenübergreifend monatlich als Teil eines Versorgungspfads; einrichtungsintern, pro Bewohnerbereich wöchentlich (bei Bedarf interprofessionell), morgendliche Blitzkonferenz pro Station täglich (Fokus Risikopatienten), interprofessionelle ethische Fallkonferenz monatlich) als Teil der regulären Arbeitszeit<br>2.2.2 Einrichtungsübergreifende gemeinsame Dokumentation in einer digitalen PHB-Akte (idealerweise mit Zugriff auf Daten der elektronischen Krankenakte), die alle PSK-relevanten Informationen enthält (Diagnosen, Befunde, vollständiger Medikationsplan incl. OCT und Nahrungsergänzungsmittel, etc.)<br>2.2.3 Entwicklung (interdisziplinärer) PSK-Versorgungspfade, insbesondere für Pflegeprobleme mit erheblichem Einschätzungsbedarf und Pflegehandlungen mit hohem Interaktionsanteil (z. B. Inkontinenz, Dekubitus, Demenz, altersangemessene Medikation), incl. Evaluationskonzept und Ergebniskontrolle
Zeithorizont:<br>*Kurz bis mittelfristig umsetzbar*		*Zu Zeithorizont: Der Abschluss regionaler Rahmenvereinbarungen zur Zusammenarbeit im Netzwerk ist bei Vorhandensein eines Organisators und interessierter Teilnehmer innerhalb von drei bis sechs Monaten erreichbar. Die Ausgestaltung ein-richtungsspezifischer Lösungen zu Erreichbarkeit und Zusammenarbeit ist je nach Komplexität der Fragestellungen in sechs bis zwölf Monaten zu erreichen. Der Ausbau telemedizinischer Ansätze ist stark abhängig von Umfang und technischer Ausgestaltung der angestrebten Lösung. Wenig aufwändige Ansätze können sofort, technisch komplexere Lösungen kurz- bis mittelfristig umgesetzt werden. Die Etablierung von Fallkonferenzen ist kurz- bis mittelfristig möglich. Die Einrichtung einer gemeinsamen digitalen Dokumentation ist in Abhängigkeit von den technischen Voraussetzungen kurz- bis mittelfristig erreich-bar. Die Entwicklung von Versorgungspfaden nimmt je nach Komplexität und Stringenz der Ablauforganisation einige Wochen bis mehrere Monate in Anspruch.*
**3. INFRASTRUKTUR DES PFLEGEHEIMS**	3.1 Auf- bzw. Ausbau einer elektronischen Infrastruktur, die sowohl eine Interoperabilität von AIS und PIS gewährleistet als auch telemedizinische Betreuung ermöglicht	3.1.1 Suffiziente (W)LAN-Anbindung<br>3.1.2 Hard- und Software für digitale PHB-Akte<br>- PHB-Akte idealerweise mit Zugriff auf Daten aus der elektronischen Krankenakte<br>- Digitale Therapieziele sollen strukturiert für Behandlungsteam, PHB und Angehörige zur Verfügung stehen<br>- Prüfung der Integration von zur PSK-Vermeidung zentraler Informationsobjekte auf Ebene der Telematikinfrastruktur (z. B. Early Warning Score, Medikationsplan)<br>3.1.3 Hard- und Software für telemedizinische Anwendungen<br>- Einfache Anwendung: Videotelefonie ermöglichen<br>- Erreichbarkeit der PHB sicherstellen (eigenes Device (Handy/Tablet/Laptop) oder einrichtungsinternes Device)<br>- Komplexere Anwendungen: Telemedizinische Diagnostik und Monitoring ermöglichen<br>- Integration von digitalen Gesundheitsanwendungen (DiGA), dabei Identifikation geeigneter DiGa’s z. B. auf Vorschlag der Pflegekräfte

Bausteine und Zeithorizont	Unterpunkte	Erläuterungen, auch zu Zeithorizont
	3.2 Schaffung einer räumlichen und medizintechnischen Infrastruktur für Diagnostik- und Behandlungsmöglichkeiten vor Ort	3.2.1 Verfügbarkeit eines Raums für Diagnostik und Behandlung<br>3.2.2 Anschaffung Medizintechnik (Sono, Röntgen, EKG)<br>3.2.3 Verfügbarkeit Labor (eigene Geräte, alternativ Kooperation mit mobilem Labor (Laborbus))<br>3.2.4 Verfügbarkeit Verbrauchsmaterial<br>3.2.5 Unterbringungskonzepte bei speziellen PSK-Risikokonstellationen
***Zeithorizont*:**<br>*Kurz bis mittelfristig umsetzbar*	3.3 Niedrigschwelligen Zugriff auf externe Evidenz ermöglichen	3.3.1 Abonnement relevanter Fachzeitschriften (analog, digital)<br>3.3.2 Einrichtung regelmäßiger interner Fortbildungen zur Diskussion aktueller Forschungsergebnisse, Leitlinieninhalte, etc.<br><br>*Zu Zeithorizont: Der Auf- bzw. Ausbau einer elektronischen Infrastruktur einschließlich Schulung des Personals ist für z. B. einfachere telemedizinische Anwendungen innerhalb von sechs bis zwölf Monaten zu erreichen. Komplexere Strukturen wie die Einführung einer digitalen Patientenakte sind mittelfristig umsetzbar. Die Schaffung einer räumlichen und medizintechnischen Infrastruktur ist abhängig vom geplanten Umfang kurz- bis mittelfristig möglich. Die Einrichtung eines niedrigschwelligen Zugriffs auf externe Evidenz ist sofort möglich.*
**4. INTERNE PROZESSE IN DEN EINRICHTUNGEN**	4.1 Einführung von Bezugspflege als Standard-Pflegesystem	4.1.1 Gesamte pflegerische Planung und Versorgung von festen PHB-Gruppen durch eine bestimmte Pflegeperson (incl. Vertretungs- und Delegationsregelungen) zur personellen Kontinuität als elementarer Bestandteil einer menschenwürdigen Pflege
	4.2 Erarbeitung von SOPs zum Umgang mit PSK-relevanten Risikokonstellationen	4.2.1 Etablierung eines initialen PSK-Risikoassessments durch pflegerisch und hausärztlich tätigen Personen einschließlich Maßnahmenplan und Auswahl des/der geeigneten PSK-Versorgungspfads/e<br>4.2.2 Standardisiertes Aufnahmemanagement<br>4.2.3 Standardisiertes Überleitungsmanagement<br>4.2.4 Ausbau der Präventionsmöglichkeiten in den Pflegeeinrichtungen (Sturz, Dekubitus, Ernährungsmanagement, Schmerzmanagement, Medikationsanalyse, Infektionsprophylaxe, Vorsorgeplanung, Überwachung Gesundheitszustand, ethische Entscheidungs-findung nach den Wünschen der Bewohner)<br>4.2.5 Umsetzung bereits bestehender Standards (z. B. der Sachverständigenstandard Sturzprophylaxe in der Pflege)
	4.3 Etablierung eines Advanced Care Planning, Bewohneranamnese	4.3.1 Einrichtung von Beratungsmöglichkeiten zu Vorsorgevollmachten, ggf. auch zu Patientenverfügungen<br>4.3.2 Vorhaltung der entsprechenden notwendigen Dokumente<br>4.3.3 Etablierung von Routinen in Absprachen mit den Bevollmächtigten<br>4.3.4 Verfügbarkeit rechtlicher Assistenz gewährleisten<br>4.3.5 Beachtung der S2k-Leitlinie „Einwilligung von Menschen mit Demenz in medizinische Maßnahmen“ bei hirnorganischen Beeinträchtigungen<br>4.3.6 Pflegeeinrichtungen können die Einführung der gesundheitlichen Versorgungsplanung gem. § 132g SGB V durch entsprechend qualifiziertes und weitergebildetes Personal mit einer anteiligen Kassenfinanzierung implementieren
	4.4 Etablierung einer qualifizierten (pflegerischen) Rufbereitschaft	4.4.1 Gestaltung des Stellenschlüssels und des Dienstplans unter Berücksichtigung der Verfügbarkeit einer qualifizierten (pflegerischen) Rufbereitschaft für Notfälle in Zeiten, in denen typischerweise mit reduzierter Besetzung zu rechnen ist (Nachtdienst, Wochenende, Feiertage)

Bausteine und Zeithorizont	Unterpunkte	Erläuterungen, auch zu Zeithorizont
	4.5 Verantwortlichkeit definieren für Initiierung und Begleitung von Veränderungsprozessen und Implementierungs-strategien	4.5.1 Benennung einer qualifizierten Person zur Verantwortlichen für die Initiierung und Begleitung von Veränderungsprozessen sowie deren Implementierung in den Arbeitsalltag (‚Change-Manager') 4.5.2 Funktion des Change-Managers muss sich in der Planung von Stellenschlüssel und Skills-Mix der Mitarbeitenden widerspiegeln
Zeithorizont: *Kurz bis mittelfristig umsetzbar*		*Zu Zeithorizont: Die Einführung von Bezugspflege ist abhängig von der Größe des zu versorgenden Bereichs. In einem kleineren Bereich ist eine Etablierung (einschließlich Vorbereitung und Erprobung) innerhalb von sechs bis zwölf Monaten zu erreichen. Die Etablierung einer qualifizierten (pflegerischen) Rufbereitschaft ist bei vorhandenem Personal sofort umsetzbar, gleiches gilt für die Festlegung der Verantwortlichkeit für die Initiierung und Begleitung von Veränderungsprozessen und Implementierungsstrategien. Die Erarbeitung von SOPs im Umgang mit PSK-relevanten Risikokonstellationen ist je nach Komplexität innerhalb von ein bis drei Monaten je SOP möglich. Die Etablierung eines Advanced Care Plannings kann kurz- bis mittelfristig umgesetzt werden.*
**5. RECHTLICHE ASPEKTE**	5.1 Neustrukturierung bestehender gesetzlicher Regelungen (SGB V und SGB XI)	5.1.1 Partielle Integration der Pflegeversicherung in die Krankenversicherung, um so die sozialrechtliche Trennung von Behandlung, Rehabilitation und Pflege zu durchbrechen
	5.2 Neustrukturierung bestehender Richtlinien zur Übertragung heilkundlicher Aufgaben für den Ausbau und die Stärkung der pflegefachlichen Kompetenz, die das deutlich veränderten Morbiditätsspektrum der PHB wiederspiegelt	5.2.1 Aufnahme von zu übertragenden heilkundlichen Aufgaben in die Heilkundeübertragungsrichtlinie, die den Anforderungen durch das veränderte Morbiditätsspektrum der PHB gerecht werden (z. B. Erweiterung des Diagnosespektrums) 5.2.2 Stärkere Berücksichtigung der Fort- und Weiterbildungsverpflichtung der Pflegeeinrichtungen zur Qualifikation von Pflegefachpersonen in heilkundlichen Fragen wie der gerontopsychiatrischen Versorgung, Wundmanagement, Schmerzmanagement etc., um die Übertragung heilkundlicher Aufgaben zu erleichtern
Zeithorizont: *mittel- bis langfristig umsetzbar*		*Zu Zeithorizont: Änderungen bestehender gesetzlicher Regelungen und Richtlinien werden sich mittel- bis langfristig umsetzen lassen.*

**6. VERGÜTUNGS-STRUKTUREN**	6.1 Abrechnungsmöglichkeiten von PSK-bezogenen Aktivitäten von Pflegeheimen gegenüber der Pflegeversicherung mit dem Ergebnis der Vermeidung von Hospitalisierungsbedarfen	6.1.1 Schulungskosten für Personal<br>6.1.2 Abschreibungen von baulichen Investitionen und Geräte-Investitionen<br>6.1.3 Mehraufwand der Netzwerkarbeit und der initialen PSK-Assessments<br>6.1.4 Setzen von kollektiv- oder selektivvertraglichen Anreizen zur Prävention bzw. Nicht-Hospitalisierung von PHB (Vermeidungsbudget)
	6.2 Experimentierräume entwickeln für neue ergebnisbelohnende Vertragsgestaltungen (Anreiz zur Reduktion Pflegeheim-sensitiver Krankenhausfälle)	6.2.1 Regionale populationsbezogene Vertragsgestaltungen mit Invest- und Ergebnisteilung<br>6.2.2 Monitoring von morbiditäts- und altersadjustierten Hospitalisierungszahlen und Veröffentlichung
	6.3 SGB XI-finanzierte wissenschaftliche Begleitevaluation	6.3.1 Kosten/Nutzen-Analysen über mehrere Jahre<br>6.3.2 Qualitative Befragungen von Angehörigen, Patienten und Pflegenden
Zeithorizont: *mittelfristig umsetzbar*		*Zeithorizont: Die Weiterentwicklung und Umstrukturierung der Vergütungsstrukturen sowie die wissenschaftliche Begleitevaluation der Effekte ist vor einem mittelfristigen Zeithorizont zu betrachten.*

Abkürzungen: ACP = Vorausplanung der Versorgung am Lebensende (Advanced Care Planning), AGnES = Arztentlastende, Gemeindenahe, E-Health-gestützte, Systemische Intervention durch qualifizierte Hausarztpraxismitarbeiter, EKG = Elektrokardiogramm, EVA = Entlastende Versorgungsassistent/-in (auch nicht-ärztliche Praxisassistent/-in genannt), PHB = Pflegeheimbewohnerinnen und -bewohner, PRISCUS = Liste mit potenziell inadäquate Medikation im Alter (Personen ab 65 Jahre), PSK = Pflegeheim-sensitiver Krankenhausfall, SGB XI = Elftes Buch Sozialgesetzbuch, SOP = Standardvorgehensweise (Standard Operating Procedure); VerAH = Versorgungsassistent/-in in der Hausarztpraxis.
